# Epigenetic consequences of toxic stress in preterm infants and their mothers: protocol for the premature infants, moms, and the social determinants of health—an epigenetic investigation study

**DOI:** 10.3389/fped.2026.1730456

**Published:** 2026-04-29

**Authors:** Kathryn Malin, Yvette Conley, Carmen Giurgescu, Roger Brown, Jessica McDonald, Tracy Heiman, Sara Anderson, Jennifer Dahlman, Rosemary White-Traut

**Affiliations:** 1 College of Nursing, Marquette University, Milwaukee, WI, United States; 2 College of Nursing, Children's Hospital and Health System Inc—Milwaukee Campus, Milwaukee, WI, United States; 3 School of Nursing, University of Pittsburgh, Pittsburgh, PA, United States; 4 College of Nursing, University of Central Florida, Orlando, FL, United States; 5 Schools of Nursing, Medicine, and Public Health, University of Wisconsin-Madison, Madison, WI, United States; 6 College of Nursing, University of Illinois Chicago, Chicago, IL, United States

**Keywords:** methylation, NICU, *NR3C1*, protocol, *SLC6A4*, stress

## Abstract

**Introduction:**

Over 10% of infants in the United States are born prematurely. Life-saving interventions in the NICU often expose these infants to stressful stimuli. When stress becomes excessive and prolonged, it is considered toxic and may disrupt brain development and physiological stress responses. Both preterm infant and their mothers are vulnerable to toxic stress from the NICU. Epigenetic changes are hypothesized to link early toxic stress exposure with long-term outcomes in preterm infants. However, research exploring maternal environmental influences and the dyadic relationship remain limited.

**Purpose:**

The Preterm Infants, Moms, and the Social Determinants of Health: an Epigenetic Investigation (PRIMS-EI) study aims to examine how maternal environmental influences and toxic stress from the NICU impact methylation of the *NR3C1* and *SLC6A4* genes in both preterm infants and their mothers.

**Methods:**

This descriptive study will include 102 preterm infant-maternal dyads. Validated instruments assess maternal environmental influences and toxic stress from the NICU. Buccal swabs are collected from both preterm infants and mothers during the first week of life and again one week before NICU discharge. Infant stress is measured daily using the Neonatal Infant Stressor Scale.

**Results and Discussion:**

Funded by an NIH K23 award, data collection is ongoing. Prior research links early toxic stress to methylation changes in infants, but maternal patterns and influences remain unexplored. This study will contribute to understanding epigenetic alterations in the dyad and their implications for future health and development.

## Introduction

Every year over 10% of all infants born in the United States (US) are delivered prematurely (prior to 37 weeks completed gestation) (1). These infants require complex, life-saving care in the Neonatal Intensive Care Unit (NICU) and costs associated with this care are estimated to be over $26.2 billion a year, not including costs associated with post-discharge care (2). While survival rates for premature infants have improved over the last 20 years, the essential life-saving interventions required while in the NICU are fraught with stressful stimuli, separation from parents, and repeated painful procedures (3, 4). While stress is a normal physiologic response to challenges, if prolonged and excessive it is considered toxic and becomes a pathologic process that alters both short and long-term health outcomes (5). Aberrant early-life stress experiences from the NICU have the potential to become more than the preterm infant's body can tolerate and thus become toxic, leading to permanent changes in brain development, function, and physiologic stress responses (5, 6). Mothers of preterm infants also experience toxic stress from the often-unexpected preterm birth of their infant, changes in parental role-attainment, the NICU environment, and the degree of infant illness (7–10). Preterm infants whose mothers report high levels of toxic stress during NICU hospitalization are more likely to demonstrate suboptimal reflexes and attention as well as increased lethargy (11). Furthermore, it is imperative that maternal toxic stress while in the NICU be considered in the context of maternal environmental influences, as infant early life stress is directly linked to their mother's environment (4, 12).

Repeated and prolonged NICU toxic stress exposures during early neurodevelopment increases the risk for changes in stress responses through epigenetic alterations in preterm infants and their mothers (13). Epigenetics is the study of phenotypic changes in gene expression that do not involve DNA sequencing. Many epigenetic alterations result from environmental exposures and may have lasting impacts on health and illness (14, 15). Specifically, epigenetic alterations are a hypothetical link between early toxic stress exposures in preterm infants and biologic changes experienced later in life (16). Epigenetic alterations in preterm infants' secondary to early life toxic stress is an emerging focus of neonatal research. Specifically, alterations in the glucocorticoid-related gene *NR3C1* and the promoter region of the serotonin promoter gene *SLC6A4* have been linked to toxic stress in both preterm infants and their mothers during the perinatal period (16–19). Inclusion of epigenetic measures connecting stress, environmental influences, and health outcomes is a research priority (20).

**Figure 1:**
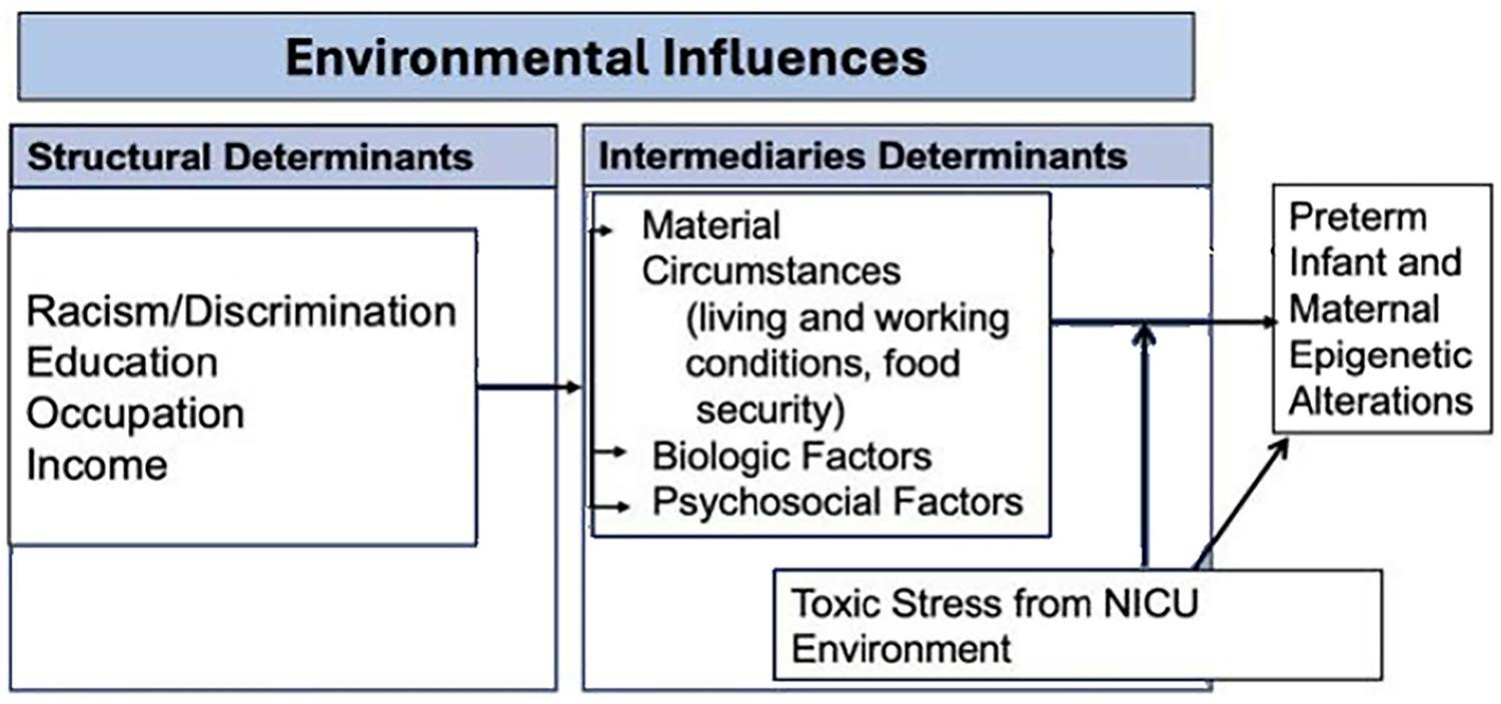
The Relationships among the environmental influences, toxic stress from the NICU environment and preterm infant and maternal epigenetic alterations.

Despite this, the evidence connecting early toxic stress from the NICU to maternal factors remain ill-defined. Furthermore, investigation into a possible dyadic relationship between the preterm infant and their mother's stress is nonexistent. While the relationship between environmental influences and epigenetic alterations has been described (13), there remains a need to identify how the dyads' environmental influences impact physiologic disruptions and adaptations. Furthermore, the potential moderating effect of toxic stress from the NICU environment warrants more detailed evaluation. How maternal environmental influences and the NICU environment impact toxic stress and epigenetic alterations remain an important missing piece in understanding the potential for preterm infant-maternal dyadic health and development. The purpose of this study is to delineate the impact of maternal environmental influences and toxic stress from the NICU environment on epigenetic alterations in preterm infants and their mothers (Figure 1). The focus of this manuscript is the presentation of the research protocol.

### Aims

The aims of this study are as follows: first to evaluate how maternal environmental influences and toxic stress from the NICU environment are associated with alterations in methylation of *NR3C1* and *SLC6A4* in preterm infants and their mothers. Secondly, to evaluate whether toxic stress from the NICU environment moderates the relationship between maternal environmental influences and methylation of *NR3C1* and *SLC6A4.* Finally, to explore preterm infant and maternal DNA methylation for concordance.

## Methods and analysis

### Design

This study employs a descriptive longitudinal design. Data will be collected from 102 preterm infant-maternal dyads at two time points. Time 1 is within the first week of life and Time 2 is one week prior to discharge from the NICU. Continuous data measuring preterm infant stress will be collected daily while in the NICU.

### Study setting

Two NICUs in the Midwest region of the US serve as the study sites. The first site is a 72-bed, level four NICU which serves a diverse population from the entire state and has an average daily census of 63. The second NICU is a 22-bed, level three NICU which serves a rural population with an average daily census of 13.

### Training of research team

This research is supported by a training grant from the National Institutes of Health (NIH) and includes three years of mentored research experiences. The primary investigator will attend structured educational offerings focused on epigenetics, toxic stress, and actor-partner modeling. Formal mentoring through collaboration, guided readings, and observational experiences is also integrated into the training plan. Finally, all members of the research team will complete the required Collaborative Institutional Training Initiatives (CITI) as well as required Research Security Policies from the NIH. Additionally, a NICU parent consultant will support the training of research staff through discussion and role playing of consent procedures and discussion scenarios that are anticipated to occur. These trainings are focused on assuring best communication practices and trauma informed communication with families of preterm infants requiring NICU hospitalization.

### Sample size and study population

The power was set to detect a moderate to large (R-squared) effect from X to change in Y_Delta (X = predictors for each person in the dyad, Y_Delta = change in % methylation) at alpha = 0.05 two-tailed test using the general rule-of-thumb. Approximately 80 preterm infant-maternal dyads using 6 predictors would provide sufficient power at 0.80 to assess Aims 1 and 2 with the understanding that N>=50 + 8 m, where *N* = sample size and m = number of predictors. Based on this power analysis and an attrition rate of 22% in our feasibility study (21), we will enroll 102 preterm infant-maternal dyads with an expected retention of 80 dyads throughout the study. Inclusion criteria for participation account for genetic relationships between the preterm infant and their mother, maternal environmental exposures, and possible birth stress. These criteria include:

- Mother speaks English or Spanish
- Mother is identified as the birth-mother (defined as the person who gave birth to the infant)
- Infant has been admitted to the NICU and is less than seven days old
- Infant was born at or less than 32 weeks gestation
- Infant will be discharged from the hospital with the birth mother
- Infant will not be transferred to another unit in the hospital prior to discharge
- Infant does not have a known genetic defect
- Infant does not have a lethal diagnosis
- In the event of multiple births (i.e., twins or triplets), one infant will randomly be chosen to participate

If the infant dies prior to completion of the study, the dyad will be excluded from any further data collection because of the potential for missing data.

### Study variables

Measurements of the independent variables align with two overarching and interconnected concepts: maternal environmental influences (operationalized as either structural determinants or intermediary determinants) and toxic stress from the NICU. The outcome variables are preterm infant and maternal epigenetic alterations. The conceptual framework of the study is displayed in Figure 1. Detailed definitions of the concepts, measures, data source, and timing of data collection are displayed in Table 1.

**Table 1:** Definitions, measures, data source, and timing of data collection.

Concept	Measures (including psychometrics)	Data Source	Time Points		
			T1	T2	Daily
Maternal Environmental Exposures (structural determinants of health occurring prior to and during pregnancy)	Discrimination: -Everyday Discrimination Scale: 10-items, self-reported measure of frequency of routine, relatively subtle discriminatory experiences in everyday social situations. *α* = .87-.92 in diverse samples of adults (22, 23) -Major Experiences of Discrimination Scale: 9-item, self-reported measures of lifetime experiences of perceived unfair treatment reflective of structural racism. α = .82-.83 in diverse samples of adults (24) -Crime Subscale of the Perceived Neighborhood Scale: 9-item, self-reported measures of experience and belief about the level of crime in their local area. α = .83-.85 in sample of parents of young children (25)	Mother self-report	x	x	
	Education level, Occupation, Income: Maternal Questionnaire: self-reported level of education completed, current occupation, and current single and household income.	Mother self-report	x	x	
Maternal Environmental Exposures (intermediary determinants of health occurring during pregnancy)	Material Circumstances: -Household Food Security Survey Module (FSSM): a two-item food insecurity screening utilizing a Likert scale in response to statements. Higher scores indicating low food insecurity. Questions include: “Within the past 12 months we worried whether our food would run out before we got money to buy more.” and “Within the past 12 months the food we bought just didn't last and we didn't have money to get more.”; α = .94 in diverse samples of adults with and without young children (26, 27) - Ross Neighborhood Disorder Scale^67^: 15-item questionnaire assessing the physical and social order in one's community, higher scores indicate higher levels of neighborhood disorder; α = .82 in samples of pregnant black women (28) - Questions assessing monthly cost for housing: Healthy People 2030 SDOH-04 “reduce the proportion of families that spend more than 30 percent of income on housing” (29)3/28/26 7:08:00 AM	Mother self-report	x	x	
	Biologic Factors: -Mother Characteristics: race/ethnicity, age, pregnancy complications (e.g., preeclampsia, gestational diabetes), history of chronic illness |-Preterm Infant Characteristics: race/ethnicity, sex, GA^b^, birthweight, Apgar scores, PMA^c^, surgical procedure, IVH^c^, NEC^d^	EHR^a^	x	x	
	Psychosocial Factors: -Edinburgh Depression Scale: a widely used 10 question screening tool to evaluate symptoms of postpartum depression. Scores of 9 or higher, or any indication of suicidal ideation requires referral for further mental health care ∝ = .66-.96 in postpartum mothers from diverse backgrounds. (30, 31) -General Anxiety Disorder-7: 7-item instrument measuring the severity of generalized anxiety disorder; cut points of 5, 10, and 15 have been established to indicate mild, moderate, and severe anxiety, α = .91 in postpartum women (32, 33) -Perceived Social Support: measures perception of social support in three domains family, friends, & social support; four items rated on a 7-point Likert Scale for the three domains; scores range between 12 & 84, with lower scores indicating lower perceived social support; = .81-.98 in samples of peripartum women (34)	Maternal Self-Report	x	x	
Toxic Stress from NICU Environment	Neonatal Infant Stressor Scale (NISS): Measures both the acute and chronic environment for individual preterm infants through quantification of daily experiences while in the NICU. 44 acute and 24 chronic stressful NICU events are scored daily and an average acute and chronic score will be calculated for the entire hospitalization; higher scores indicate higher acute and chronic neonatal stress secondary to their environment; α = .87 in NICU patients (35)	EHR, Maternal Self-Report^a^	X	x	x
	Parental Stressor Scale—NICU: measures the parental experience of the NICU environment and the stress that results from the (1) sights and sounds, (2) appearance and behaviors of the infant, (3) impact on the parents' role and relationship with the baby, and (4) the parents' relationship and communication. α = .89-.90 in parents of infants in the NICU (34)	Maternal Self-Report			
Epigenetic Alterations	% DNA methylation of *NR3C1* and *SLC6A4*: infant and maternal DNA using Isohelix DNA Buccal Swabs and analysis using bisulfate conversion and next-generation sequencing.	Infant and mother buccal swab	x	x	

a EHR, electronic health record, ^b^GA, gestational age at birth, ^c^PMA, postmenstrual age at discharge, ^c^IVH, intraventricular hemorrhage, ^d^NEC, necrotizing enterocolitis.

T1, first week of life in NICU; T2, within one week prior to NICU discharge home; Daily, every day the infant is hospitalized in the NICU.

### Data collection and management

After a member of the research team obtains informed consent, data will be collected during the first week of life (T1), one week prior to NICU discharge (T2), and the daily calculations of preterm infant stress using the Neonatal Infant Stressor Scale (NISS) will be used to calculate an average acute and chronic NISS score for the entire hospitalization. Instruments used to measure the study variables are outlined in Table 1. All instrument data will be collected and stored in REDCap (36). Buccal swabs will be obtained using Isohelix swab kits (Isohelix, United Kingdom). The swabs will be inserted into the infant and mother's mouth and rubbed firmly against the inside of the cheek for a minimum of 20 s. Swabs will then be stored in either a −20 or −80 degree freezer (location dependent) until shipment of samples for analysis, which will occur once all samples are collected. Differences in freezer temperature does not impact methylation patterns (37). Analysis will include extraction of DNA from the buccal swabs. Next, bisulfate sequencing will be utilized to evaluate site-specific DNA methylation. Bisulfate conversion of the DNA followed by prosequencing to determine the extent of methylation across one regulatory region for each gene. Epitect Bisulfite Kis (Qiagen Corp) will be used to convert unmethylated cytosines to uracils. PCR will be used to generate overlapping fragments for sequencing. Sequencing will be performed, sequences aligned, and percent methylation established for each methylation site. To ensure scientific rigor and obtain robust, reliable and unbiased results, samples with incomplete bisulfite conversion or poor sequencing will be removed from analysis. Data quality checks will also include 10% technical replicates for each assessment.

### Data analysis

Data analysis will be facilitated using MPLUS and NCSS (38, 39). Missing data will be imputed using multiple imputation based on missing completely at random assumption, with tests for validation of this assumption. Descriptive exploratory analyses will be completed using distribution statistics. To evaluate how maternal environmental exposures and toxic stress are associated with alterations in methylation of *NR3C1* and *SLC6A4* in preterm infants and their mothers we will describe the relationship in alterations in DNAm of *NR3C1* and *SLC6A4* and the extent that maternal environmental influences and toxic stress from the NICU environment explain variations in DNAm changes. We will utilize bivariate regression modeling of the dyadic data to estimate the extent to which maternal environmental exposures and toxic stress from the NICU environment predict changes in DNAm of *NR3C1* and *SLC6A4* in both members of the preterm infant-maternal dyad. The two measures of NICU environment will then be evaluated as possible moderating variables between maternal environmental exposures and epigenetic alterations. Finally, to explore for preterm infant and maternal DNAm concordance, we will employ Actor-Partner Interdependence Modeling. Covariates, including varied gestational age at birth and time of data collection, will be managed through adjustments using propensity score weighting. Generalized Boosted Modeling will be used to estimate the propensity score by considering multiple covariates simultaneously. If after covariate balancing there remains unbalanced covariates, a doubly robust weighting approach will be employed (40, 41).

### Treatment or intervention

No study-related interventions are planned. All infants will receive care according to the NICU's standards of care.

## Anticipated results

We hypothesize worse maternal environmental influences will be associated with increased altered methylation patterns of *NR3C1* and *SLC6A4* for preterm infants and their mothers at T1 and T2. We also anticipate preterm infants and mothers with higher scores of toxic stress from the NICU environment will be positively associated with alterations in methylation patterns of *NR3C1* and *SLC6A4* at T2. An alternate outcome would be that mothers with both high toxic stress from the NICU environment and more negative environmental influences do not display the similar positive alterations in methylation of *NR3C1* and *SLC6A4* as mothers with high toxic stress from the NICU environment but more positive environmental influences. This outcome would be significant, possibly demonstrating a difference in the impacts of stress associated with chronic life environments and situational stress associated with NICU hospitalization. We also expect preterm infants and their mothers who require longer NICU hospitalization will demonstrate higher percentages of change in methylation of *NR3C1* and *SLC6A4* from T1 to T2. Finally, we expect preterm infants and mothers to demonstrate concordance, predicting reciprocal influences within the dyad.

## Discussion

This research provides an innovative approach to connecting maternal environmental influences and toxic stress from the NICU environment to epigenetic alterations in preterm infants and their mothers. Furthermore, this study accomplishes the necessary first steps in determining the cumulative impact of toxic stress for preterm infant-maternal dyads. There is a significant need to optimize preterm infant-maternal health outcomes and identifying relationships between toxic stress and epigenetic alterations offers a unique and meaningful new approach. No previous research has measured DNAm of *NR3C1* and *SLC6A4* in both preterm infants and their mothers at the beginning and the end of their NICU hospitalization. Additionally, this research accounts for the complex interactions between toxic stress from maternal environmental influences and toxic stress from the NICU environment, providing a comprehensive framework of toxic stress and its biomechanistic effects.

While this protocol has many strengths and is a necessary first step in describing the complex relationships among maternal environmental influences, toxic stress from the NICU and epigenetic alterations in preterm infant-maternal dyads, limitations are recognized. The decision to measure DNAm of *NR3C1* and *SLC6A4* is based on previous research describing significant relationships between early toxic stress from the NICU and epigenetic alterations in the promotor regions of genes responsible for regulating serotonin, dopamine, and cortisol (17, 42). Despite the previous research, the results from this study will be limited in scope and future epigenome-wide association studies (EWAS) are needed to provide genome-wide variations. Another limitation is the use of the NISS to measure toxic stress exposures while in the NICU. Though the NISS is a validated instrument (42), inherent limitations associated with scale include the use of the EHR to quantify NICU activities which may be incorrectly charted, no evaluation of the infants' responses to the environment, and poor concurrent validity with other measures of stress (43, 44). Furthermore, this protocol does not include measurement of possible protective factors against stress or physiological markers of toxic stress while in the NICU. Possible physiological markers of stress that should be considered in future studies include cortisol in breast milk, markers of inflammation in saliva, or changes in heart rate. Including all infants born before 32 weeks' gestation improves feasibility and focuses the sample on those classified as “very preterm” and “extremely preterm”. However, there remains important developmental differences within these two groups which may also account for alterations in DNAm and must be considered when interpreting results. Finally, additional covariates, such as maternal gravida/para status, that may impact maternal stress responses and are not currently accounted for should be considered in the analysis.

Despite these limitations, this study offers a novel assessment of toxic stress in preterm infant-maternal dyads. It is the first to conceptualize preterm infants and their mothers as an integrated dyadic unit within the context of toxic stress. The anticipated results are expected to yield foundational evidence to inform future research on toxic stress, epigenetic alterations, and subsequent health outcomes in this vulnerable population.
